# Physiotherapists’ perspectives on barriers to implementation of direct access of physiotherapy services in the United Arab Emirates: A cross-sectional study

**DOI:** 10.1371/journal.pone.0253155

**Published:** 2021-06-11

**Authors:** Arwa Alnaqbi, Tamer Shousha, Hamda AlKetbi, Fatma A. Hegazy

**Affiliations:** 1 Physiotherapy Deparment, Kuwait Hospital, MOHAP, Dubai, UAE; 2 Department of Physiotherapy, College of Health sciences, University of Sharjah, Sharjah, UAE; 3 Faculty of Physical Therapy, Department of Physical Therapy for Musculoskeletal Disordered and its Surgery, Cairo University, Giza, Egypt; 4 Physical Medicine & Rehabilitation Department, Rashid Hospital, DHA, Dubai, UAE; 5 Faculty of Physical Therapy, Department of Physical Therapy for Growth and Development Disorders in Children and Its Surgery, Cairo University, Giza, Egypt; Prince Sattam Bin Abdulaziz University, College of Applied Medical Sciences, SAUDI ARABIA

## Abstract

**Background:**

There are two primary ways of accessing physiotherapy for service users around the world. The direct access, as opposed to the indirect access which requires a referral from a general physician, has several merits including better quality, timeliness, cost effectiveness of treatment and better probability of preventing acute conditions from turning into chronic ailments. Despite these benefits, several countries including the UAE, do not allow direct access to physiotherapists. This study aims to understand the level of awareness among practicing physiotherapists in the United Arab Emirates (UAE) about direct access and to determine whether any of their demographic variables influence the way they perceive the concept. Further, the study sought to explore the perceived barriers and benefits of direct access according to the participating physiotherapists.

**Subjects and methods:**

An observational cross-sectional study was employed. The questionnaire survey developed by Bury and Stokes in 2013 was adapted and employed in this study. The instrument had six sections with close-ended items using a Likert five-point scale to rate them. Two hundred and sixty-four physiotherapists answered the questionnaire shared with them through a web link. Finally, MANOVA was employed to explore any influence of demographic variables on the opinions of the respondents.

**Results:**

The findings showed that 70% of participants were aware about direct access while nearly 30% were completely unaware. Younger physiotherapists were more willing to endorse the practice whereas older ones were more apprehensive of the barriers. The main barriers reported were the limited support from the physicians and policy makers, professional autonomy, and the limited scope of practice for the physiotherapists, as well as evidence-based practice. The impact of demographic variables on direct access indicated that physiotherapists under the age of 23 endorsed direct access more strongly than other age groups.

**Conclusion:**

More efforts are needed to implement direct access in the UAE, considering the benefits of improved professional status, cost savings, patient satisfaction, and higher efficiency. This study recommends leadership support, professional autonomy, and mentorship as possible ways to achieve this goal.

## Introduction

Direct access is the legal right to receive and seek the evaluation, examination, and intervention by physiotherapist without the requirement of physician referral [1]. Though direct access has been gaining popularity around the world, several countries including the UAE have not yet allowed its implementation by public health services. Such measures have prevented the benefits of implementation, such as a reduction in the time required to meet with a physiotherapist, greater control over the frequency and scheduling of visits, and cost effectiveness for the patient and the public healthcare system [2]. Previous evidences indicates that recovery time for patients who approached their physiotherapists through direct access is also less [3–5].

Additionally, direct access has benefits associated to physiotherapy practice. When allowed to function as regular physicians, physiotherapists gain more professional recognition and respect [6]. As a result, many professional associations are advocating for the practice, including the World Confederation for Physical Therapy (WCPT) and the American Physical Therapy Association (APTA) with its Vision 2020 program. The APTA has named direct access and autonomous practice as its main areas of focus. In countries like the US, the dual form of government has acted as a barrier, with several states choosing not to allow direct access to physiotherapy [7]. Several medical professionals’ organizations are also opposed to it, like the American Medical Association, the American Academy of Orthopedic Surgeons, the American Orthotic and Prosthetic Association, and the American Academy of Orthotists and Prosthetists. According to these organizations and their member physicians, there are still concerns of patient safety and competence of the physiotherapists to make an able diagnosis.

Direct access for physiotherapy services may be an important step in supporting patient-centered model of care in the UAE, in allowing equitable access to the best treatments, and in allowing physiotherapists to fully engage with the medical community as full-fledged professionals working at their highest potential. There have been arguments that direct access would undercut the relationship among physiotherapists and other healthcare practitioners who formerly would have served as referral sources. However, this has not been the case in many other professional areas where practitioners make decisions based on referrals from other independent professionals to establish and maintain their practices. Published literature on direct access has to date primarily been limited to studies from the Netherlands, Australia, United States, and United Kingdom, with a growing body of literature as well as policy support to support its clinical effectiveness, implementation and cost-effectiveness [8–10]. In countries where patients are not allowed to access physiotherapy directly, the physiotherapists report feelings of frustration and low self-esteem [11]. This limitation also affects their ability to think critically and reason. Further, their competence is also affected as the opportunity to participate in and employ evidence based practice becomes rare. Moreover, physiotherapists also report a lack of rapport-development with clients.

Despite these benefits, no existing literature is available about UAE physiotherapists’ perspectives on direct access. It is not known whether these practitioners are aware of what direct access models allow, how supportive they are of moving to a direct access model, or how aware they are of any barriers to implementing such a model, be at the patient, provider, system, or societal level. This study, therefore, aimed to understand awareness of direct access, benefits, and barriers according to practicing physiotherapists in the UAE. The research objectives include were as follows:

1. To determine the perspectives/awareness of the physiotherapists regarding the implementation of direct access of physiotherapy services in the United Arab Emirates.
2. To examine the perceived benefits and barriers during the implementation of direct access in the UAE.

## Materials and methods

### Study design and participants

An observational cross-sectional study design was followed in the current study. The current study had been conducted between 1^st^ March 2020 and 1^st^ April 2020 (4.5 weeks). Convenient sampling [12] method was chosen where the sample was taken from a group of people who were easy to contact or to reach.

Questionnaires were sent to 300 respondents through emails and a web-based link using Google Surveys. Physiotherapists from the public and private sectors of the UAE with at least one year of working experience were included in the study. All physiotherapy students and physiotherapists with less than a year of experience were excluded from the study. The data collection in the current study was stopped after data saturation was achieved (264 respondents) with a response rate of 88%.

### Measures and instruments

The survey was adapted from Bury and Stokes (2013) [6] which was developed to enquire the opinions of World Confederation for Physical Therapy (WCPT) members about direct access to physiotherapy services. The survey included the following sections (S1 Appendix):

### Socio-economic characteristics

Section 1 The demographic details of the respondents included their gender, age, qualifications, organizational affiliation, job title, years of experience, work environment, and area of practice.

### Survey

The finalized version of the survey included the following sections:

Section 2 (**Awareness**) enquired about the respondents’ awareness of direct access and its application in physiotherapy with six items.
Section 3 (**Endorsement**) elicited their views about direct access and whether they were in favor of implementing it or not with 5 items.
Section 4 (**Obstacles/Barriers**) enquired about the barriers that may affect the implementation of direct access in the UAE and asked them to provide examples of eleven such possible barriers.
Section 5 (**Perceived Benefits**) enquired about the perceived benefits of direct access using six items detailing the various benefits portrayed in existing literature. An open-ended question allowing the respondents to share any other benefits that may occur to them.
Section 6 (**Expected Benefits of Various Resources**) included 10 items and focused on expected benefits of the implementation of direct access to guide the evidence-based practice of the physiotherapists. The survey was tested for its reliability and validity in the UAE population prior initiation of the study.

### Procedures

The Web-based version of the questionnaire was developed on the google forms containing 48 questions and subcategories. It was then sent to the respondents starting from 1^st^ March 2020 over the course of 4.5 weeks. A pilot test was undertaken to assess the accuracy and clarity of the survey involving 20 physiotherapists. The major purpose of performing the reliability test was to evaluate the logistics of the method and identify any kind of concerns with the language. Furthermore, feedback attained during the pilot test informed survey modifications, and the final version of the survey was determined following several consensus meetings. Content validity of the instrument was improved by eliciting experts’ feedback about the questionnaire while the pilot study helped in establishing its internal reliability. Cronbach’s alpha was used to establish the internal consistency between the various scale items and was observed to be 0.87 which is above the recommended value of 0.70 [13].

A Google Survey link was created to be shared via email with the physiotherapists along with a cover letter informing them about the study and eliciting their consent to participate in it. The email had a personalized active web link that directed the respondent directly to the questionnaire without using any type of password. The nonrespondents received a phone call and a reminder e-mail after a period of two weeks with some questionnaires necessitating a second reminder as well, both using the digital mailbox. All collected questionnaires were processed, checked for incomplete entries, and stored in a storage drive for safety. The questionnaire entries were entered into an Excel file which was later converted into an SPSS file for data analysis.

### Statistical analysis

All entries were transferred into an IBM SPSS version 22 file which was used for data analysis (IBM SPSS Statistics for Windows, Version 22.0. Armonk, NY: IBM Corp.). Missing entries were first confirmed. Eight missing values were identified among the 264 cases, so they were replaced by the Mean values, as the recommendation allows for up to 10% of the total entries to be replaced in such manner [14].

Descriptive statistics were used to analyze demographic data, including the frequency distribution, percentile, mean, and standard deviation, in order to summarize, and draw conclusions. Normality of data distribution was investigated using Shapiro-Wilk test which revealed normal distribution of data. Multivariate analysis of variance (MANOVA) was employed to explore any influence of demographic variables on the opinions of the respondents.

The minimum sample size (n = 230) was calculated using G*Power 3.1.9.4 software (Heinrich-Heine-Universität Düsseldorf, Düsseldorf, Germany; http://www.gpower.hhu.de/) to detect an effect size of 0.06 (obtained from our unpublished pilot study), with a power of 0.90, and alpha level set at 0.05. A total sample of 264 participants was recruited assuming 10–20% drop out rate.

### Ethics approval

An information sheet and an informed consent form were available on the first page of the questionnaire. Therefore, a written electronic consent was obtained. Participants were free to withdraw at any time without giving explanations and no personal identification was requested to retain information confidentiality. Participants were given no incentives for participating in the questionnaire. The system of Google Forms only provides responses for questionnaires with 100% completion rate. The present study followed the ethical code for a web-based research and conforms to the principles embodied in the Declaration of Helsinki. This study was approved by the research Ethics Committee of University of Sharjah, UAE [Approval number: REC-20-06-18-02-S]. The current study was reported in accordance with the STROBE checklist for the observational studies (S2 Appendix).

## Results

The demographic profile of the respondents revealed that more female (57.2%) than male (42.8%) therapists participated in the survey (Table 1). Respondents were predominantly from the group of 33–42 years old (58.7%) and 23–32 years old (24.6%). Baccalaureate degree (60.2%) was the most popular qualification followed by 36.4% Master’s and only 2.7% doctorate holders. Most respondents worked with Ministry of Health and Prevention (MOHAP) (31.1%), while Dubai Health Authority (DHA) (18.2%), the private health sector (16.7%) and the Abu Dhabi Health Services Company SEHA (11.4%) were also represented. More than half of the sample had a senior role (57.2%) though 37.9% were working as juniors and a few as academicians (3.8%). Most participants had 6–10 years of experience (31.8%) followed by 29.5% with 11–15 years and 19.7% with 1–5 years of working experience.

**Table 1:** Demographic profile of the respondents (n = 264).

Demographic Variables	Frequency	Percentage
**Gender**		
**Male**	113	42.8%
**Female**	151	57.2%
**Age**		
**Less than 23 years**	14	5.3
**23–32 years**	65	24.6
**33–42 years**	155	58.7
**43–52 years**	22	8.3
**More than 52 years**	8	3
**level of education**		
**Baccalaureate**	159	60.2
**Master degree**	96	36.4
**DPT**	2	.8
**PhD**	7	2.7
**Main Work Setting**		
**MOHAP**	82	31.1
**DHA**	48	18.2
**Health Authority Abu Dhabi**	30	11.4
**DHA**	20	7.6
**Private Health Sector**	44	16.7
**Academic Field**	14	5.3
**Other**	26	9.8
**Job Title**		
**Junior**	100	37.9
**Senior**	151	57.2
**Academic**	10	3.8
**Consultant**	3	1.1
**Years of Experience**		
**1–5**	52	19.7
**6–10**	84	31.8
**11–15**	78	29.5
**16–20**	31	11.7
**21–25**	10	3.8
**More than 25 years**	9	3.4
**Work Environment**		
**Outpatient Setup**	77	29.2
**Inpatient Setup**	29	11
**Home Care**	8	3
**Academic Institution**	14	5.3
**Others**	2	0.8
**Multiple forms**	134	50.8
**Current Area of Practice**		
**General Practice**	55	20.8
**Cardiopulmonary Rehabilitation**	3	1.1
**Geriatric/Neurologic**	20	7.6
**Wellness/Health promotion**	1	0.4
**Education**	6	2.3
**Sports/Musculoskeletal**	26	9.8
**Research**	1	0.4
**Pediatric**	20	7.6
**Others**	132	50

MOHAP: Ministry of Health and prevention; DHA: Dubai health authority; DPT: Doctor of Physical Therapy Degree; PhD: Doctor of Philosophy in physical Therapy.

Before analyzing the findings, data was checked for any missing values or wrong entries. Eight missing values were identified among the 264 cases, so they were replaced by the Mean values, as the recommendation allows for up to 10% of the total entries to be replaced in such manner [14]. Table 2 shows the Mean and Standard Deviations observed for each of the items in the questionnaire scales to understand which aspects of Direct Access influence the opinions of the physiotherapists more than the others.

**Table 2:** Statement analysis.

Factor	Item	Statement	Yes (%)	No (%)	Not Sure (%)
**Awareness of Direct Access**	1.	Do you have theoretical knowledge of direct access in physical therapy?	71.6	17	11.4
	2	Are you aware of current practices in direct access in physical therapy?	68.6	18.9	12.5
	3	Do you understand the concept of direct access in physical therapy?	75	8.7	16.3
	4	Do you agree that direct access simply means when patients are referred to physical therapist by a physician?	77.3	8.7	14
	5	Do you agree that direct access will help in eliminating delays in the provision of effective care to patients?	81.8	3	15.2
	6	Have you read any journals, articles, reports or any publications on direct access/self-referral?	39.8	56.8	3.4
			**Mean**	**Standard Deviation**	
**Endorsement of Direct Access**	1	Do you agree to support this type of model, if it were to be implemented in the UAE?	4.36	.847	
	2	Do you agree to practice this type of model, if it were to be implemented in the UAE?	4.42	.776	
	3	Do you agree that policy makers are in favor of direct access/self-referral?	3.23	1.2	
	4	Do you agree that the patients/clients/public are in favor of direct access/self-referral?	3.95	.918	
	5	Do you agree that doctors/physicians are in favor of direct access/self-referral for physical therapy?	2.7	1.21	
**Barriers to Direct Access**	1	Medical views (i.e., Doctors/Physician perspectives)	3.76	1.087	
	2	Lack of evidence-based practices	3.4	1.17	
	3	Prolonged Waiting lists/service demand	3.16	1.16	
	4	Scope of practice of PT	3.5	1.28	
	5	Lack of professional autonomy of PT	3.67	1.23	
	6	Payment/ reimbursement model (Insurance scheme)	3.23	1.13	
	7	Laws and regulations	3.97	1.20	
	8	Entry-level PT education	3.3	1.17	
	9	Professional skills/competencies of PTs	3.38	1.20	
	10	Views of service users i.e., patients believe and trust	3.14	1.09	
	11	Self-perception/ Image of PTs	2.88	1.2	
**Perceived Benefits**	1	More efficient access to outpatient PT services	4.4	.812	
	2	Improved patient satisfaction	4.36	.878	
	3	Improve the efficiency of resource utilization (e.g., radiology)	4.36	.852	
	4	Improved professional status for physical therapists	4.52	.935	
	5	Healthcare system savings by preventing acute conditions from becoming chronic	4.48	.859	
**Expected Benefits from Resources**	1	Published relevant literature describing the evidence of direct access safety, cost effectiveness and long-standing models that utilize direct access in clinical educational resources /database or educational materials from trusted organizations	3.76	1.18	
	2	Consultation services	3.73	1.11	
	3	Attending a conference, workshop, and trainings on direct access / Access to Continuing Professional Development (CPD) /electronic resources	3.87	1.13	
	4	Mentorship from someone experienced working in a direct access system	3.93	1.17	
	5	Lack of professional autonomy of PT	4.06	1.11	
	6	Payment/ reimbursement model (Insurance scheme)	3.56	1.11	
	7	Handout explaining direct access for patients	3.55	1.13	
	8	Leadership support	4.15	1.17	
	9	Infrastructure setup	3.62	1.17	
	10	Integration with Primary Health Centers (PHC) to help in sending patient	3.79	1.11	

MANOVA was employed to assess the impact of demographic variables on direct access. Gender, qualifications, work settings, work environment, and areas of practice were not related to endorsement, awareness, benefits, and barriers of direct access. Age, however, influenced the perceptions of benefits, barriers, and endorsement of direct access though with low effect size (Table 3). Physiotherapists under the age of 23 endorsed direct access more strongly than others, while the 33-42-year-old group perceived more benefits. Finally, the oldest physiotherapists in the 43–52 years group believed the most in direct access’s benefits. A similar relationship existed with job titles, where the academicians and consultants endorsed direct access more willingly while the juniors perceived more barriers than others for the implementation of direct access. Senior physiotherapists believed the most in the perceived benefits of direct access. Similarly, the physiotherapists with 21–25 years of experience endorsed direct access and believed the most in its perceived benefits.

**Table 3:** MANOVA between age groups for direct access (n = 264).

Dependent Variable	F	*Df*	Significance	Partial Eta Squared	Estimated Marginal Means	Mean values
Endorsement	4.068	4	.003*	.059	Less than 23	21.14
					23–32 years	17.48
					33–42 years	18.84
					43–52 years	19.09
					More than 52 years	19.38
Barriers to Direct Access	4.783	4	.001*	.069	Less than 23	27.79
					23–32 years	37.65
					33–42 years	38.15
					43–52 years	37.68
					More than 52 years	36.5
Perceived Benefits	2.878	4	.023*	.043	Less than 23	21.2
					23–32 years	21.14
					33–42 years	22.50
					43–52 years	23.59
					More than 52 years	20.38

df: Degree of freedom; F: F value P value: probability value (P<0.05)

*: statistically significant at level of (P<0.05).

## Discussion

This study aimed to understand the opinions of practicing physiotherapists in the UAE about the grant of direct access to their practice. Earlier research has shown that direct access has several benefits for physiotherapists while their awareness, knowledge, attitudes, and behavior towards this construct leads to improved outcomes. The research purpose was to confirm if this was true for the physiotherapists practicing in the UAE. Our results indicated that even though 71.6% of respondents were aware about direct access, there was a large section of physiotherapists who, by virtue of being unaware about the term and its significance were unlikely to endorse it and become advocates for its implementation. This issue has also been acknowledged by other studies in different countries [6,15,16]. At the same time, among those physiotherapists who knew about direct access, its endorsement was high (Mean 4.42) which further emphasizes that improving the awareness of direct access is a priority.

Among other results, gender was not found to influence any of the direct access variables, a finding that has been reported by Holdsworth and Webster in the year 2004 [17]. Age, on the other hand, was found to be associated with growing appreciation for the benefits of direct access, except for the youngest physiotherapists under the age of 23 who endorsed it most strongly. The 33–42 years old group believed that the barriers are strong, revealing that with more experience and age, physiotherapists are acquiring a more realistic view of the situation which makes them aware of more benefits but also more barriers against the implementation of direct access. The academicians and consultants were stronger endorsers of direct access because they have better theoretical appreciation of its benefits while the juniors perceived the barriers to be stronger. In line with the results of the older and more experienced (21–25 years) physiotherapists, the senior physiotherapists perceived the most benefits of direct access.

The barriers of direct access were limited support from the physicians, the policy makers, limited professional autonomy, limited scope of practice, and the absence of evidence-based practice. On the other hand, self-belief, patients’ trust, and long service lists and demands were not believed to be strong barriers. These results have ample support from studies conducted elsewhere, which shows that a global response and demand for direct access and steps taken at the macro level can be of immense benefit to the cause [6,18–21]. However, there is an important consideration required to achieve the desired outcome: the belief in the competence of the physiotherapists has to be established with more studies along the lines of Aiken and McColl [22] and Moore et al [23]. These studies have shown that the diagnostic ability and the agreement between the treatment endorsed by physiotherapists and physicians are quite comparable if not better for the physiotherapists. Such studies can help further the cause of direct access.

**Fig 1:**
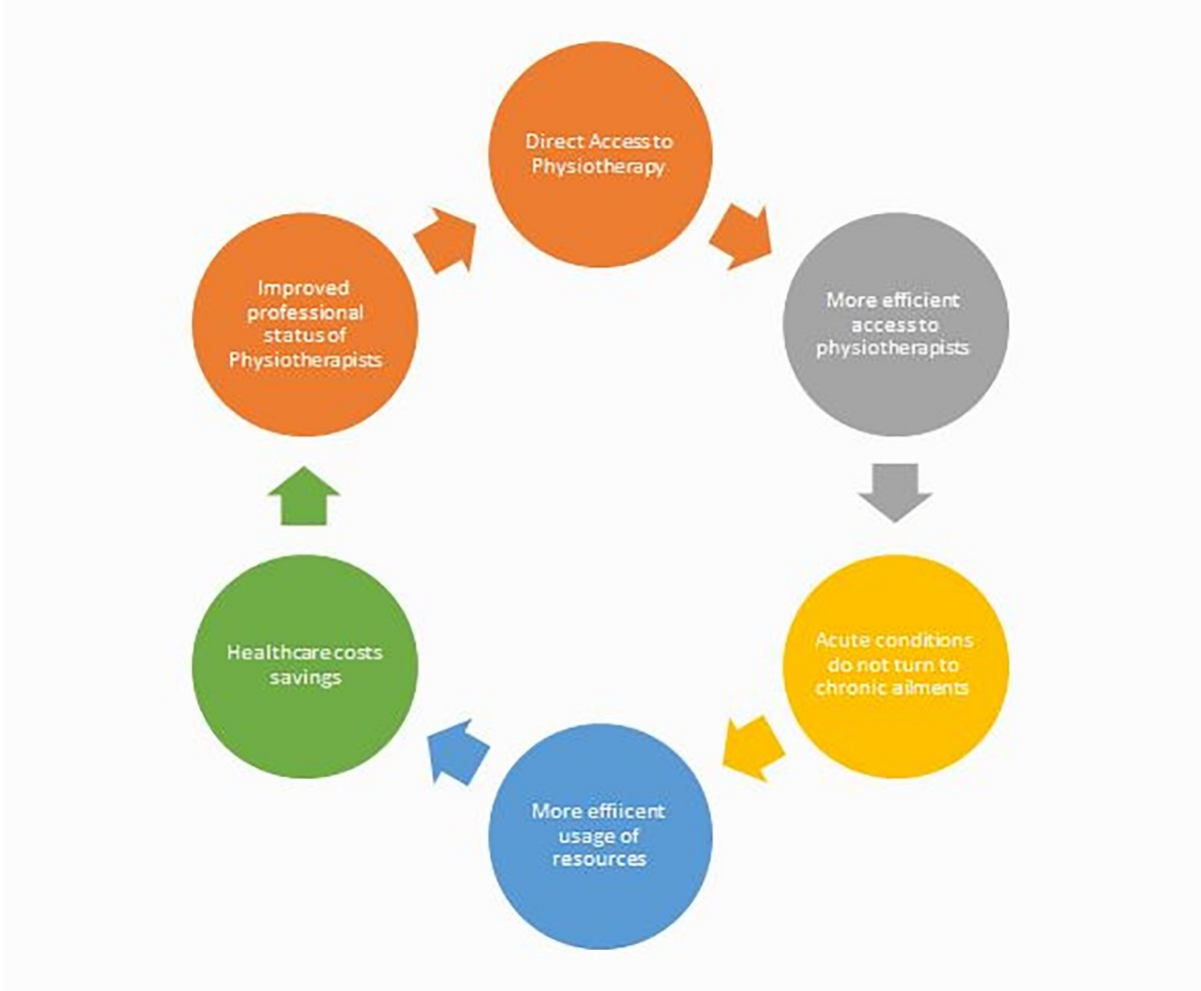
Benefits of direct access.

Among the perceived benefits of direct access, some were improved professional status for the physiotherapists, cost savings for healthcare, and more efficient services (Fig 1) [1,4].

Finally, the physiotherapists considered the leadership support, professional autonomy, and the mentorship support as the most helpful resources for leveraging the benefits of direct access. These benefits also resonate with earlier studies [1,4,24,25]. Other resources included attending conferences and workshops on the topic, training, electronic resources, and other published literature.

## Limitations

Some limitations affected the course of this research. First, the sampling could not be conducted randomly due to the nature of the study. Qualitative data could not be captured in the study as the researchers believed that being one of the first studies on the subject in the UAE, a cross-sectional quantitative design was better suited. However, the lack of rich qualitative data is recommended for future researchers. Some of the sources which could not be included for their opinions of direct access are the institutional leaders, the patients, and the physicians.

## Recommendations

In response to these results, several recommendations can be drawn. Awareness of direct access in the UAE can only grow once physiotherapists have a combined presence on professional platforms where they can discuss, lobby, and reflect on the changes desired by them. At present, only 314 of the 2866 practicing physiotherapists in the country are members of the Emirates branch of WCPT [26]. Physicians and policymakers who are against direct access have to become engaged in dialogues to help them understand that physiotherapists can ably diagnose and treat several ailments. On the other hand, emerging evidence indicates that direct access physiotherapy can offer improved outcomes in terms of quality of life, disability, and healthcare costs compared to primary physician-led medical care. [27] Similarly, service users should also be educated about direct access and its benefits, as this would help gain their trust and turn them into allies in this quest. The results of physiotherapists perceiving more barriers for direct access with age, seniority, and experience is a serious lapse and should be addressed as it indicates that they are feeling disheartened about gaining professional autonomy which may have repercussions in the quality of their care as well. The acceptability and affordability of direct access has to be explored in detail using studies that enquire about the perceived benefits and barriers of direct access from other stakeholders like physicians, policymakers, and patients themselves. The insights collected from such studies will help decision-makers to further gain support for direct access and address the remaining concerns. Finally, the benefit of engaging various partners in dialogue will manifest in better synergy and other incremental benefits as healthcare operates in a multi-disciplinary practice and gains a lot through improved communication. Similarly, Igwesi-Chidobe [16] highlighted the need to ensure effective communication between healthcare professionals, and with patients, clarity on the physiotherapy scope and the direct access pathway, and adequate resources to meet the required demand. The acceptability and affordability of direct access has to be explored in detail using studies that enquire about the perceived benefits and barriers of direct access from other stakeholders like the physicians, policy makers, and the patients themselves. The insights collected from such studies will help decision-makers to further gain support for direct access and address the remaining concerns. Finally, the benefit of engaging various partners in future dialogue will manifest in better synergy and other incremental benefits as healthcare operates in a multi-disciplinary practice and gain a lot through improved communication.

## Clinical implications

The findings of this study indicate support for applying direct access hold value for the healthcare practice in the country. As direct access has not been examined before in the context of the UAE, this study’s findings enrich its theoretical background and support evidence-based practice. By showing that there is a keen need to bring all stakeholders associated with physiotherapy; the policymakers, physicians, patients, caregivers, general public, and the physiotherapists themselves on the table to discuss the implementation of direct access, this study has supported multidisciplinary approach to clinical practice.

## Conclusions

This study has shown that awareness of direct access among physiotherapists working in the UAE is high. Better organization and promoting awareness will lead to improved representation and advocacy for direct access. Considering the benefits of direct access for the profession, the patients, and the healthcare in general, immediate steps should be undertaken to allow physiotherapists to access their patients directly. Further, this study has important implications for the profession of physiotherapy globally. Direct access should be granted to physiotherapists even in the developed countries which shows that their capabilities are still doubted. This study adds to the increasing body of evidence which argues that allocating direct access to the physiotherapists is a win-win equation for the patients, the professionals, and the community at large.

## Supporting information

S1 Appendix Questionnaire. (PDF)

S2 Appendix STROBE checklist. (DOC)
