# Correction: Physiotherapists’ perspectives on barriers to implementation of direct access of physiotherapy services in the United Arab Emirates: A cross-sectional study

**DOI:** 10.1371/journal.pone.0314067

**Published:** 2024-11-14

**Authors:** Arwa Alnaqbi, Tamer Shousha, Hamda AlKetbi, Fatma A. Hegazy

There are errors in the Author Contributions. The correct contributions are:

**Conceptualization:** Arwa Alnaqbi, Tamer Shousha, Fatma A. Hegazy.

**Data curation:** Arwa Alnaqbi, Hamda AlKetbi.

**Formal analysis:** Arwa Alnaqbi, Tamer Shousha, Hamda AlKetbi, Fatma A. Hegazy.

**Investigation:** Fatma A. Hegazy.

**Methodology:** Arwa Alnaqbi, Tamer Shousha, Hamda AlKetbi, Fatma A. Hegazy.

**Project administration:** Arwa Alnaqbi, Fatma A. Hegazy.

**Resources:** Arwa Alnaqbi, Hamda AlKetbi, Fatma A. Hegazy.

**Software:** Hamda AlKetbi.

**Supervision:** Arwa Alnaqbi, Tamer Shousha, Fatma A. Hegazy.

**Validation:** Arwa Alnaqbi, Tamer Shousha, Fatma A. Hegazy.

**Visualization:** Arwa Alnaqbi, Hamda AlKetbi, Fatma A. Hegazy.

**Writing–original draft:** Arwa Alnaqbi, Fatma A. Hegazy.

**Writing–review & editing:** Tamer Shousha, Fatma A. Hegazy.
