# Supplementary material for: Physiotherapists’ perspectives on barriers to implementation of direct access of physiotherapy services in the United Arab Emirates: A cross-sectional study
Source: PLoS One. 2021 Jun 11;16(6):e0253155. doi: 10.1371/journal.pone.0253155 (PMC8195403; doi:10.1371/journal.pone.0253155)
Supplement: S1 Appendix — (PDF) [file pone.0253155.s001.pdf]

Questionnaire

Section I: Demographics

1. Which of the following best describes your gender? Select one.
- ❑ Male
- ❑ Female

2. What is your age? Select one.
- ❑ < 23 years
- ❑ 23-32 years
- ❑ 33-42 years
- ❑ 43-52 years
- ❑ > 52 years

3. What is your highest physical therapy qualification? Select one.
- ❑ Baccalaureate
- ❑ Master
- ❑ DPT
- ❑ PhD or other doctoral

4. Which of the following best describes your main work setting?

- ❑ Ministry of Health and Prevention (MOHAP)
- ❑ Department Health Authority (DHA)
- ❑ Health Authority Abu Dhabi
- ❑ Dubai Health Authority (DHA)
- ❑ Private Health Sector
- ❑ University/ College 'Academic'
- ❑ Other Sector (Please Specify) ……………………………………….

5. What is your job title for your main work setting?

   Kindly specify ....

6. For how many years have you been in practice? Select one.

- ❑ 1-5
- ❑ 6-10
- ❑ 11-15 years
- ❑ 16-20 years
- ❑ 21-25 years
- ❑ > 25 years

7. Which of the following best describes your place of work environment? Select all that apply

- ❑ Outpatient setup
- ❑ Inpatient setup
- ❑ Patients home/Home care
- ❑ Academic institution
- ❑ Others , please specify...

8. What is your current area of practice? (you can choose more than one answer)

- ❑ General practice
- ❑ Geriatric/Neurologic
- ❑ Education
- ❑ Sports/Musculoskeletal
- ❑ Pediatric
- ❑ Cardiopulmonary rehabilitation
- ❑ Wellness/ Health promotion
- ❑ Community development
- ❑ Research

others: ______________________________

Section II: Awareness of Direct Access

1. Do you have theoretical knowledge of direct access in physical therapy?

- ❑ Yes
- ❑ No
- ❑ Not sure

2. Are you aware of current practices in direct access in physical therapy?

- ❑ Yes
- ❑ No

- ❑ Not sure

3. Do you understand the concept of direct access in physical therapy?

- ❑ Yes
- ❑ No
- ❑ Not sure

4. Do you agree that direct access simply means when patients are referred to physical therapist by a physician?

- ❑ Yes
- ❑ No
- ❑ Not sure

5. Do you agree that direct access will help in eliminating delays in the provision of effective care to patients?

- ❑ Yes
- ❑ No
- ❑ Not sure

6. Have you read any journals, articles, reports or any publications on direct access/self-referral?

- ❑ Yes
- ❑ No
- ❑ Not sure

Section III: Views on direct access/self-referral					
**Questions/Response**	**Strongly agree**	**Agree**	**Neutral**	**Disagree**	**Strongly disagree**
1. Do you agree to support this type of model, if it were to be implemented in the UAE?	1	2	3	4	5
2. Do you agree to practice this type of model, if it were to be implemented in the UAE?	1	2	3	4	5

3. Do you agree that policy makers are in favor of direct access/self-referral?	1	2	3	4	5
4. Do you agree that the patients/clients/public are in favor of direct access/self-referral?	1	2	3	4	5
5. Do you agree that doctors/physicians are in favor of direct access/self-referral for physical therapy?	1	2	3	4	5

Section IV: Obstacles to Direct Access Implementation

In your opinion what do you think are the barriers to introducing and implementing direct access/self-referral services in the UAE?

Please tick all that apply and indicate how much impact does that barrier has, from scale 1–5, where 1 indicates small/little impact and 5 indicates huge impact on direct access to PT.

Barriers					
1. Medical views (i.e. Doctors/Physician perspectives)	1	2	3	4	5
2. Lack of evidence-based practices	1	2	3	4	5
3. Prolonged Waiting lists/service demand	1	2	3	4	5
4. Scope of practice of PT	1	2	3	4	5
5. Lack of professional autonomy of PT	1	2	3	4	5
6. Payment/ reimbursement model (Insurance scheme)	1	2	3	4	5
7. Laws and regulations	1	2	3	4	5
8. Entry-level PT education	1	2	3	4	5
9. Professional skills/competencies of PTs	1	2	3	4	5
10. Views of service users i.e. patients believe and trust	1	2	3	4	5
11. Self-perception/ Image of PTs	1	2	3	4	5

Section V: Perceived Benefits

Do you agree that implementing direct access for physical therapy would result in any of the following?

Benefits/Response	Strongly agree	Agree	Neutral	Disagree	Strongly disagree
1. More efficient access to outpatient PT services	1	2	3	4	5
2. Improved patient satisfaction	1	2	3	4	5
3. Improve the efficiency of resource utilization (e.g. radiology)	1	2	3	4	5
4. Improved professional status for physical therapists	1	2	3	4	5
5. More efficient access to primary care or family practice physician service	1	2	3	4	5
6. Healthcare system savings by preventing acute conditions from becoming chronic	1	2	3	4	5

Kindly describe in your own words other suggestions of benefits below:

Section VI: Expected Benefits of Various Resources for Direct Access Implementation

If direct access were to be implemented, please rate how important the following resources would be of benefit.
Please tick all that apply and indicate the importance of the resources 1–5, where 1 is less importance and 5 is very important.

Resources					
1. Published relevant literature describing the evidence of direct access safety, cost effectiveness and long-standing models that utilize direct access in clinical educational resources /database or educational materials from trusted organizations	1	2	3	4	5
2. Consultation services	1	2	3	4	5
3. Attending a conference, workshop, and trainings on direct access / Access to Continuing Professional Development (CPD) /electronic resources	1	2	3	4	5
4. Mentorship from someone experienced working in a direct access system	1	2	3	4	5
5. Lack of professional autonomy of PT	1	2	3	4	5
6. Payment/ reimbursement model (Insurance scheme)	1	2	3	4	5
7. Handout explaining direct access for patients	1	2	3	4	5
8. Leadership support	1	2	3	4	5
9. Infrastructure setup	1	2	3	4	5
10. Integration with Primary Health Centers (PHC) to help in sending patient	1	2	3	4	5

Survey adapted from: Tracy J. Bury, Emma K. Stokes, A Global View of Direct Access and Patient Self-Referral to Physical Therapy: Implications for the Profession, Physical Therapy, Volume 93, Issue 4, 1 April 2013, Pages 449–459, https://doi.org/10.2522/ptj.20120060 with permission from WCPT
